# Impact of common skin diseases on children in rural Côte d’Ivoire with leprosy and Buruli ulcer co-endemicity: A mixed methods study

**DOI:** 10.1371/journal.pntd.0008291

**Published:** 2020-05-18

**Authors:** Rie Roselyne Yotsu, Colombe Coffie Comoé, Germaine Taïba Ainyakou, N’guessan Konan, Amari Akpa, Aubin Yao, Julien Aké, Bamba Vagamon, Rigobert Abbet Abbet, Roger Bedimo, Roderick Hay

**Affiliations:** 1 School of Tropical Medicine and Global Health, Nagasaki University, Nagasaki, Japan; 2 Department of Dermatology, National Center for Global Health and Medicine, Tokyo, Japan; 3 Department of Tropical Medicine, Tulane University School of Public Health and Tropical Medicine, New Orleans, United States of America; 4 Department of Social Sciences, University of Felix Houphouët Boigny (UFHB), Abidjan, Côte d’Ivoire; 5 Laboratoire d’Étude et de Recherches Interdisciplinaire en Sciences Sociales (LERISS), Abidjan, Côte d’Ivoire; 6 Department of Social Sciences, University of Peleforo Gon Coulibaly, Korhogo, Côte d’Ivoire; 7 MAP International Côte d’Ivoire, Abidjan, Côte d’Ivoire; 8 Raoul Follereau Institute Côte d’Ivoire, Adzopé, Côte d’Ivoire; 9 Department of Dermatology, Université Alassane Ouattara, Bouaké, Côte d’Ivoire; 10 Ministry of Health, Abidjan, Côte d’Ivoire; 11 Division of Infectious Diseases, University of Texas Southwestern, Dallas, United States of America; 12 International Foundation for Dermatology, London, United Kingdom; Swiss Tropical and Public Health Institute, SWITZERLAND

## Abstract

**Background:**

Skin-related neglected tropical diseases (skin NTDs) occur against a background of a very high prevalence of common skin diseases in sub-Saharan Africa. In this study, we examined the knowledge, attitude and practices (KAP) and the impact of common skin diseases in children living in a leprosy and Buruli ulcer (BU) co-endemic district in a west African country of Côte d’Ivoire, in order to help inform disease control efforts for skin NTDs.

**Methods and principle findings:**

Fourteen focus group discussions (FGDs) with schoolchildren, 5 FGDs with parents of a child affected with skin disease(s), and 27 in-depth semi-structured interviews with key personnel were conducted. The Children’s Dermatology Quality of Life Index (CDLQI) questionnaire was applied to 184 schoolchildren with skin diseases. We found that there was ignorance or neglect towards skin diseases in general, due to their high prevalence and also the perceived minimal impact on children’s daily lives. While the median score for the CDLQI questionnaire was 5 (IQR 2–9) out of 30, a range of scores was observed. Symptoms such as pruritus and experiencing bullying by classmates contributed to reduction in their quality of life. Poor hygiene was considered as a major cause of skin diseases.

**Conclusions/Significance:**

Despite their high impact on affected populations, we observed a high level of ignorance and neglect toward common skin diseases. There is a critical need to increase awareness of skin diseases, or skin health promotion, which supports changing of the health-seeking behaviour for skin conditions. This will aid in early detection and treatment of the skin NTDs, in addition to providing benefits for those affected by other skin diseases. Educational opportunities should be utilized to their utmost. One would be associated with water, sanitation, and hygiene (WASH) strategies, but careful messages need to be developed and delivered.

## Author summary

Areas endemic for skin-related neglected tropical diseases, or skin NTDs, also show high prevalence rates for other skin diseases. It is important to gauge the understanding of the people’s knowledge, attitudes and practices towards these more common skin diseases as well as their impact. This study was conducted in communities of the western African country, Côte d’Ivoire where there is co-endemicity of skin NTDs, including leprosy and Buruli ulcer. Ignorance and neglect of skin diseases in general were observed, due to the high prevalence and also their perceived minimal impact on children’s daily lives. However, some children were shown to have been affected significantly by their skin disease through our interviews and questionnaires. Our study results suggest a critical need for awareness raising activities, or skin health promotion, which supports changing health seeking behavior for skin conditions to aid in early detection and treatment of the skin NTDs. This may also be advantageous for those affected by other skin diseases. Messages need to be developed with care during delivery of education to avoid creating negative images of diseases, and further, stigma and discrimination.

## Introduction

There is currently an initiative towards integration of the skin-related neglected tropical diseases, or skin NTDs, under the guidance of the World Health Organization (WHO) and their global partners [1]. Skin NTDs comprise a sub-group of NTDs which share a common feature, skin involvement, and disease control strategies are in the process of being structured in order to leverage this feature. Many countries in sub-Saharan Africa are co-endemic with different combinations of skin NTDs including leprosy, Buruli ulcer (BU), yaws, lymphatic filariasis, onchocerciasis, and mycetoma along with scabies which was recently added to the NTD group in 2017 [2].

The west African country of Côte d’Ivoire is co-endemic for a number of skin NTDs. In particular, it is one of the most endemic countries for BU, accounting for 36% of the 61,119 globally reported cases since reporting to WHO started in 2002 [3]. According to the statistics of the National Program for the Control of BU, over 40% of new BU cases detected has been in children under the age of 15. It is also listed as one of the 22 global priority countries for leprosy control by WHO, as it reports approximately 1000 new cases per year, amongst which close to 10% of cases are found in children [4]. These children affected by either disease, with lack of appropriate care and in the late stages of their disease with disabilities and deformities, are often forced to leave school and deprived of educational opportunities [5, 6].

Interventions targeting children should therefore be a part of strategies for early diagnosis and treatment of children affected by these disabling diseases, and targeting a common population with a single intervention, is likely to be effective and efficient. However, for many of the integrated activities for skin NTDs, for example active case detection, it is evident that many other common and non-NTD skin diseases will also be encountered. The prevalence of common skin diseases in children is extremely high in sub-Saharan Africa and reported prevalence ranges from 26.7 to as high as 80.4% [7–12]. Early case detection of skin NTDs can potentially be hampered by such high prevalence of common skin diseases as this may add a significant workload to activities and a need for extra resources, including diagnostic methods and treatment [13]. In order to succeed in integrated activities, it is also necessary to have a good understanding of the knowledge base, attitudes, and practices of individuals in the community toward the more common skin diseases, for which only a limited number of studies exist at present. Furthermore, as children—an already vulnerable population—are the most affected, a better understanding of their relative effect on children’s health and well-being is needed in order to design interventions that decrease the impact of skin diseases amongst this population. Also, a comparative assessment of the impact of the common skin diseases and skin NTDs in affected communities has not been previously done.

The objectives of the current study were therefore 1) to delineate the knowledge, attitudes, and practices toward common skin diseases in children in NTD-endemic communities and 2) to understand the impact of skin diseases on children through interviews and by applying the children’s dermatology quality of life index (CDQLI) questionnaire, in rural Côte d’Ivoire. The knowledge, attitudes, and practices were also assessed for leprosy and BU as the study sites chosen were co-endemic for these two skin NTDs, and so that our findings will have practical implications in formulating disease control measures for skin NTDs.

This study was performed prior to the school-based skin survey project which we implemented in the same study site, and described previously [13].

## Methods

The investigation followed a qualitative approach with focus group discussions (FGDs) and semi-structured in-depth interviews, and quantitative approach using the CDQLI questionnaire.

### Ethics statement

Written informed consent was obtained before participants were enrolled in the study. For schoolchildren, written informed consent was obtained from their parents or guardians. The purpose, procedures, and data confidentiality were explained by school teachers / directors, or community health workers (CHWs). All names of participants were made anonymous by applying identification codes during data analysis and reporting. Ethical approval was granted by the ethics committees of the Ministry of Health (Côte d’Ivoire) and the National Center for Global Health and Medicine (Japan).

### Study area and participants

The study was conducted in the health district of Adzopé, a southern part of Côte d’Ivoire as described previously [13]. We identified five villages from this district which had previously reported cases of leprosy and Buruli ulcer as suitable for the study: Bouapé (population 4,615), Ahouabo (7,071), Ananguié (9,238), Miadzin (3,570), and Grand Akoudzin (4,013).

Children with skin diseases at the time of the study were identified from the primary schools in these five villages. Among those identified, children in the 2^nd^ year of Basic Course (CE2; equivalent to 4^th^ grade in US/UK school system) were purposively selected for the FGD interviews. Beside the schoolchildren, key persons defined as individuals who would either have a strong influence on children (parents, teachers, leaders) or have direct or indirect experience of dealing with children’s skin diseases (healthcare providers, traditional healers) were also interviewed.

### Data collection

Data collection took place during May-June 2014. FGDs were conducted for schoolchildren and parents so as to create group dynamics and generate rich discussion. A total of 14 FGDs were performed for children. Because of an assumption of differences in knowledge and attitudes depending on parents’ occupation, separate FGDs were performed for children of farmers (n = 7) and children of civil servants (n = 7). Additionally, 5 FGDs were performed for parents (farmers and civil servants combined). Each FGD consisted of 6 to 10 participants. For the key persons other than the parents, semi-structured in-depth interviews taking into account the differences in their professional backgrounds were designed. A total of 27 interviews were conducted. Interviewees included community health workers (n = 4), primary school teachers (n = 4), nurses at primary health clinics (n = 2), a doctor (n = 1), traditional healers (n = 6), and leaders (community, religious, women and youth associations) (n = 10).

Interview topic guidelines were used to moderate the interviews (Topic guidelines S1). The guidelines included open-ended questions that allowed respondents to express themselves freely with questions relating to knowledge of skin diseases, how they experienced the diseases, effects on their daily lives, and their attitudes towards others with skin diseases. In addition, questions about leprosy and BU were specifically asked to determine the respondents’ knowledge, attitudes, and practices towards these two skin NTDs. Photographs of randomly chosen common skin diseases were provided to the schoolchildren in order to facilitate the interviews.

**Fig 1:**
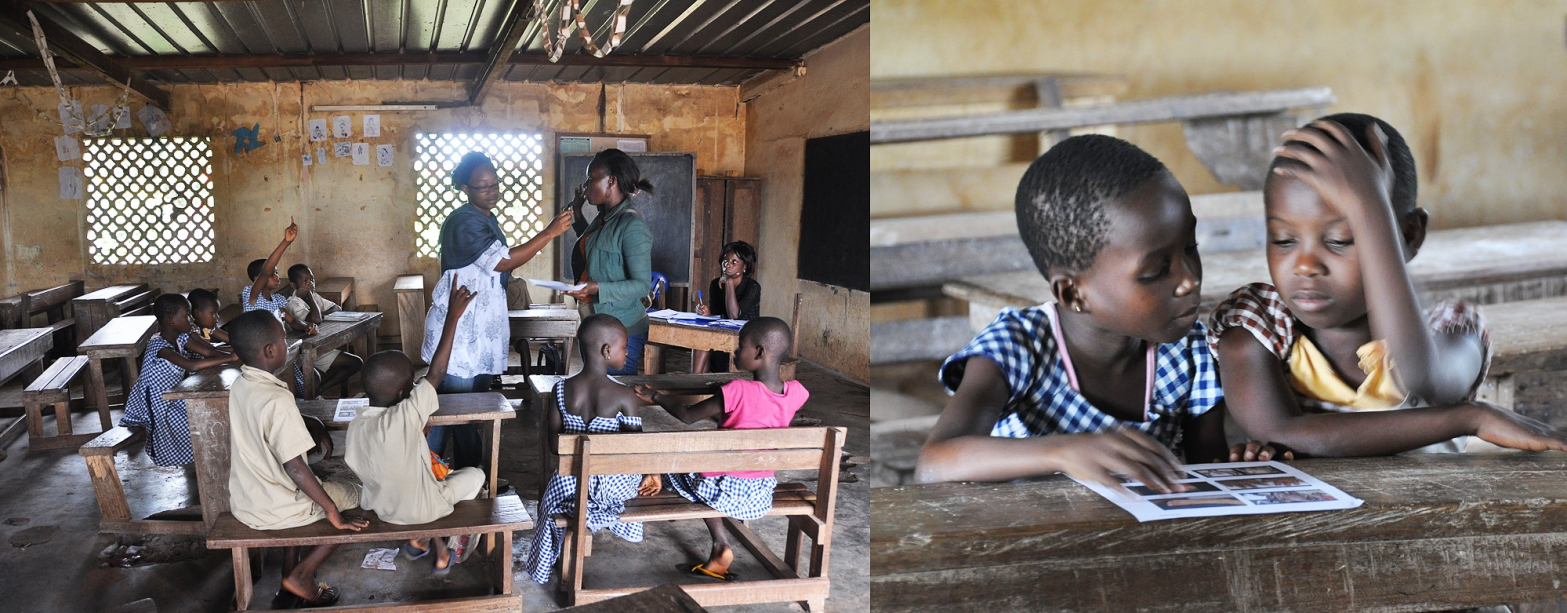
Scene of focus group discussion with schoolchildren.

FGDs were held in a quiet classroom for durations ranging from 30 minutes to 1 hour. Tables and bench chairs were arranged in a ‘U’ shape to enable visibility and interaction between the facilitator and the students/parents (Fig 1). In-depth interviews were held depending upon the respondents’ availability, but in a quiet room. Interviews were mainly conducted in French (official language and language taught in schools) and led by two sociologists specialized in qualitative research methods (CCC, ATG). When the interviewee only spoke the local language, a translator was invited to facilitate the interview. Interviews were recorded using a voice-recorder, after obtaining consent. Recruitment and interviews were conducted until the study reached a saturation point [14].

**Fig 2:**
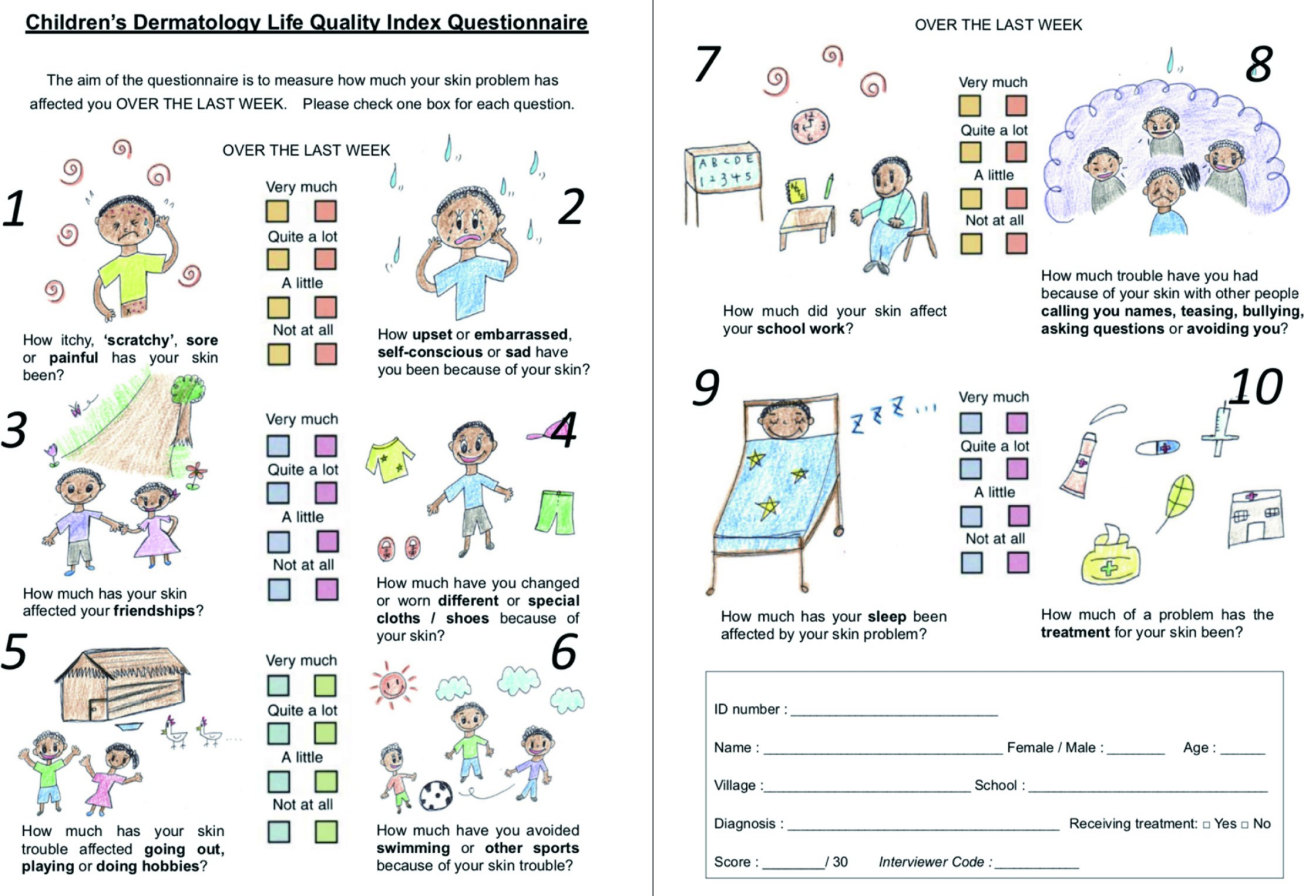
Modified Children’s Dermatology Life Quality Index (CDLQI) questionnaire [English version].

We translated the Children’s Dermatology Life Quality Index (CDLQI) (the cartoon version) by Holme *et al*. from English to French, and modified the illustration of a pet dog to those of children to increase familiarity and also to fit the African context [15] (Fig 2; S1 Fig; S1 Table). The tool was first pilot-tested in CE2 level schoolchildren in Abidjan, the economic capital city, and the language was revised where necessary by CCC and ATG (S1 Table) before we applied the tool to children who participated in the FGDs and to whom we further identified with skin diseases at our study site. To avoid over-sampling of certain diseases and to ensure cover for a wide variety of skin diseases, quota sampling was used in recruiting our participants. Since there was no data on distribution of skin diseases at our study site, we screened all children in the first six classes (one class from each level) to obtain an estimated prevalence of the common skin diseases in schoolchildren. Based on this data, we calculated the quota with the target sample size of 200 respondents and derived a maximum number of respondents to be recruited for the top five most common skin diseases, as summarized in S2 Table. Otherwise, we recruited all those diagnosed, including BU and leprosy, and consented to our study. The diagnoses of skin diseases were made by two dermatologists.

A total of 10 questions relating to six dimensions of quality of life were asked: (i) symptoms and feelings (questions 1 and 2), (ii) leisure (questions 4, 5, and 6), (iii) school (question 7), (iv) personal relationships (questions 3 and 8), (v) sleep (question 9), and (vi) treatment (question 10). Answers were scored from 0 to 3 (0: Not at all, 1: A little, 2: Quite a lot, 3: Very much), giving a total maximum overall score of 30.

### Data analysis

All voice-recorded interview data (19 FDGs and 27 in-depth interviews) were transcribed verbatim by CCC and ATG. Interviews in local languages (n = 2) were translated into French. Analysis was performed based on the framework approach [16]. During the process of transcribing and analyzing the transcripts, key ideas, concepts, and themes were identified and categorized. The framework for coding was developed by RRY, CCC, and ATG, with the consensus of all investigators. Coding of data was performed using the MAXqda software version 11 (VERBI GmbH, Marburg, Germany). Silence, hesitation, and other meaningful body gestures were documented in brackets. The frequency of coded segments and relations between the segments were examined through text and diagrams. RRY, CCC, and ATG checked for consistency, similarity and diversity within and across the emergent themes. All processes were conducted in French so as not to lose the essence of data.

Data for the CDLQI questionnaire were analyzed using a JMP version 14.2 software (SAS, Cary, North Carolina, USA). Descriptive data are shown as n (%) for categorical variables and medians (interquartile range) for quantitative variables. Wilcoxon-rank sum tests were performed to compare CDLQI scores between groups. Severity stratification was defined as described elsewhere: 0–1: no effect on QoL; 2–6: small effect; 7–12: moderate effect; 13–18: very large effect; 19–30: extremely large effect [17].

## Results

### PART 1: Results from the interviews

A total of 52 schoolchildren (29 boys, 23 girls) aged between 7 and 14 (median 11 years, IQR 10–12) participated in our FGDs. For the FGDs with parents, a total of 30 individuals (18 males, 12 females) aged between 28 and 50 (median age 35 years, IQR 30–45) participated, of whom 17 (57%) were farmers and 13 (43%) were civil servants.

Through our analysis, a total of 8 themes were identified with 123 sub-issues. Key findings within each theme are provided below with illustrative quotes.

#### Recognition/identification of skin problems

The knowledge of skin problems by schoolchildren stems from experiences lived and shared with their family and peers. According to them, the pimples or “buttons” that appear on the body, white / dry spots or “dartre”, and itchy skin oftentimes ascribed to scabies or “gale” were frequently experienced skin problems. Defining, identifying, and recognizing a skin problem was not difficult for schoolchildren because they have been victimized because of them one or more times. The status of their parents (farmers vs. civil servants) did not seem to influence the knowledge base of the students. Some teaching sessions at schools also contributed to their overall knowledge as skin problems and the need for good personal hygiene, formed part of the students’ curriculum. This then facilitated the identification of skin problems.

In general, key persons interviewed (parents, teachers, community and religious leaders, traditional healers) recognized and defined skin diseases, citing everyday life as the basis for their knowledge. Almost all of the parents surveyed reported that at least one of their children had experienced a skin problem.

#### Interference in social relationships causing emotional distress

Skin diseases tend to change the nature of the social relationships that bind the patient to his/her peers, and children affected by skin diseases were subjected to some degree of mockery, rejection, or marginalization. For instance, students with scalp ringworm were often given nicknames and mocked for having “100 francs / 500 francs” (referring to the round lesions as pieces of coins of local currency stuck on their head), “maze”, or “white head”. Some children stayed at home to avoid being bullied. Mocking resulted in a lowering of self-esteem of an affected child in some cases, and these said that they were “feeling bad” or “feeling sad” when they had skin diseases.

‘*I feel sad*, *my friends make fun of me…They laugh at me and they say that I should not come play next to them or they will catch too*.*’ (Civil servant’s child*, *Bouapé)*

And sometimes it also caused behavioural change such as isolation, self-restraint, and self-stigma.

‘*I cannot play with my friends because I'm ashamed of this*, *I could not play with my friends*, *I think they'll have it too*?*’ (Farmer’s child*, *Bouapé)*

‘*Indeed*, *we see that*, *children who have ringworms*, *for example*, *are mocked by their peers*, *and for that*, *they are frustrated and they do not want to integrate into the group either to play*, *or to learn to read and write at school*. *They prefer not to come to school*, *so they play a lot*.*’ (School director*, *Ahouabo)*

#### Impacts of skin diseases on the states of well-being

Skin diseases with pruritus often created an uncomfortable sensation, sleep disturbance, and in some cases, this had a significant impact on the students’ academic performance. Pruritus became the object of their whole attention, and the students had difficulty in concentrating in classes.

‘*I scratched all my skin*, *I cannot go to sleep*, *my mom will tell me to sleep as that’s necessary*, *then I say that I can’t*. *Every night*, *I sit down and then I start to scratch*.*’ (Civil servant’s child*, *Miadzin)*

‘*When I start studying and then my body starts scratching*, *it stops me from studying*. *When I was at CE1 [1*^*st*^ *grade]*, *I had bad grades and it made me repeat the CE1*.*’ (Farmer’s child*, *Bouapé)*

Despite the impact of being mocked as described above, there was little motivation to hide their skin symptoms. For instance, they did not attempt to cover affected areas with clothing. However, this may have been due to the hot climate and unavailability of more covering clothing.

#### Skin diseases affecting children perceived as insignificant

Many parents regarded skin diseases as insignificant as they did not appear to affect their children’s daily activities very much and most viewed them as not being life-threatening. Many parents treated their children with traditional remedies such as leave poultices at home or creams obtained at the pharmacy.

‘*When we notice such problems on the children’s skin*, *we find it insignificant*. *Most often*, *parents take the leaves to treat ringworms and so forth*, *and then after that*, *it disappears*.’ *(Parent*, *Bouapé)*

Very few had taken the children to see a doctor. That lack of perceived importance was often associated with lack of awareness of their own children’s health challenges, such as skin diseases prevalent amongst their children.

‘*Well*, *first*, *there is ignorance*. *There is ignorance*. *For a child who has a small patch or a small button on the body*, *he says it is*, *it is a normal fact*, *and then it's trivial as well*. *There is this ignorance*. *Two*, *there is the fact that [we] do not have a health center*. *Three*, *there is also parents have no means to think a little*, *a button on the body of her child is not permanent*, *there are better things to do*. *It's when the buttons become big enough and the child cannot stand*, *the parent says*, *ah her child*, *her son is sick*. *We must take the child to the hospital*. *But*, *otherwise euhh*, *buttons are common today*, *the one in the village is trivial*. *It is that common here*.*’ (School director*, *Miadzin)*

On the other hand, almost all parents were aware that chickenpox and measles were contagious and thus they prevented their children from going to school when affected. Lack of awareness also existed among children; when not bothered by pruritus or bullying, there was a tendency to dismiss the presence of a skin disease.

#### Poor hygiene considered a major cause for skin diseases

Most adults expressed their concern about the hygienic condition of the children. Many children swam in dirty water and then just went to bed without washing, or they did not change their clothes often enough: only once or a few times a week. Adults often attributed the development of various skin diseases–including leprosy and BU–to behaviours such as these that might have an impact on health. Improving personal hygiene and increasing awareness were perceived as potentially beneficial in having an impact on children’s skin conditions.

Other measures perceived as having a potential for improving skin conditions included more awareness of types of skin diseases, screening of children’s skin, and having a health center in areas closer to where they live.

‘*Yes*, *we must educate those children for them to be clean*, *because those skin diseases come from the fact that some children do not wash themselves often enough*. *There you have it*, *one must educate in that way in regards to washing clothes regularly and washing themselves with clean water and soap*, *especially to not go bathe with dirty water*. *Well*, *hey*, *there is a dam here*, *often children go swimming and then they get dysentery everywhere they piss blood [schistosomiasis] and all that*. *Here*, *we must raise awareness of the danger what those waters contain*.*’ (Parent*, *Grand Akouzin)*

#### Preconceived images of leprosy and BU

Leprosy was often described by children because of its limb and eye manifestations, as a disease that “cuts the fingers and feet” or causes “short hands and feet, and spoiled eyes”. In their local language Attié (one of the Akan ethnic group language), leprosy was called “kokobé”, which means “pity” in English. There was very little understanding about the cause of leprosy and its transmissibility. Many children believed that the disease is contagious and they could contract it from dirty places and garbage or from playing with an affected person. No child mentioned transmission from “blood”, while some adults believed it was hereditary because they see multiple patients within the same family, or from other myths such as “curse” or “bad deeds”.

BU was often described as a disease that gives “big, swollen wounds” that also “smells bad”. However, often they seemed to attribute any large ulcer to this condition. This is reflected in the name for BU in Attié: “wépion”, which is translated as “amputated foot” in English. As was the case with leprosy, there was very little understanding of the causes of BU and its transmissibility. Some however mentioned that they could contract the disease if they had “fun in dirty water” or from mosquitos. Adults mentioned that BU is a “new disease” that was brought to their region recently, while leprosy was an “old disease”.

While the knowledge about the more prevalent skin diseases came from everyday life, knowledge of leprosy and BU was derived from various sources including family members, teachers, television, radio, newspapers, and church. Some schoolchildren had family members or neighbors who had been affected by either disease, in which case, they gave a clear description of the features. A few teachers revealed that they use leprosy or BU as examples of the result of poor hygiene.

‘*I sometimes especially in the afternoon take students with buckets*, *water for washing*, *and force them to wash in school; because we are in Africa*, *with us here it's hot*, *we are in a class where we have 40–50 students in the class*, *the heat generated by each child including one that will release is the teacher*, *is suffocating…I ask the children to wash*, *when I see that he has not washed in the afternoon*, *or it is dirty*, *“Out*! *Go wash and you come back”*. *…Well*, *how do I talk to the children of leprosy*? *I share a banal example*, *I say good when you do not wash and dirty*, *after you have buttons*, *these buttons there will evolve*, *evolve and become lesions until one notes that it is leprosy*, *so the one who does not wash he gets leprosy*.*’ (School director*, *Miadzin)*

#### “Rejection” and “pity” towards people affected with leprosy and BU

Leprosy and BU are sources of social disqualification. Many children considered both leprosy and BU to be “contagious”, “bad”, and “dangerous” diseases and reported that they could “spoil a man”. It seemed that fear of being contagious was more associated with the feeling of rejection than with their appearance. The feeling of rejection was less evident with BU than with leprosy. Rejection due to BU was mainly based on the lesions’ foul smell and many children said that they did not want to go close to a patient.

Among adults, there was a feeling of “shame” and “pity” for people affected with leprosy and BU, and at the same time, some revealed their feelings of rejection. This rejection manifested itself by verbal rejection (insult, abuse) and exclusion from the social environment, *i*.*e*. refusal to share the same social space (peer meeting, ceremony, etc.).

‘*If you see it*, *you cannot eat oh*. *It smells like that*. *I saw it well*, *well*, *well*. *It smells like that*. *You cannot even eat*.*’ (Parent*, *Ahouabo)*

‘*If there is someone who has Buruli ulcer here*, *we don’t go to his home*. *It looks like the man*, *he is rotten even*. *That’s why we set him apart*.*’ (Traditional healer*, *Bouapé)*

Others explained that a minimal level of rejection existed, and that it was more the patients who were self-stigmatizing themselves.

‘*They have much shame*, *guys hide it*, *I do not know why guys hide*, *so no it's not because people are laughing too*. *…he [patient with leprosy] eats with his neighbors*, *he drinks from the same cup as his neighbors on this side and there is no problem*, *but people are ashamed to say they have these diseases*, *that's all*.*’ (Community health worker*, *Ahouabo)*

However, the true extent of stigma and discrimination in the community was difficult to assess.

Some children linked leprosy to “witches” due to their physiological and sometimes frightening deformity. This state of affairs is also based and rooted deeply in the social concept amongst the population concerning “witches”. There is a common belief that scornful people are witches in the communities.

‘*It is said that she is a witch because she has leprosy on her*, *and in her life*, *she can never succeed*.*’ (Farmer’s child*, *Bouapé)*

One child who had a grandmother with leprosy mentioned that her grandmother was called a “witch” and was not respected within the community.

#### Medical pluralism rooted in the customs and challenges faced by the people in seeking care

Diversity was observed for the routes of treatment seeking and practices adopted for skin diseases in our study population. This was structured around six components: self-medication (plants, cosmetics, or over-the-counter medications), traditional medicine, allopathic treatment, Chinese medicine, mixed treatment, or lack of any form of treatment. For those who sought for treatment, the most utilized was traditional medicine as this has been the custom in Africa for a long time and because Africans seek firstly to find the cause of their abnormalities in exogenous factors. Traditional healers have an important reputation in their social environment for their ability to treat diseases through supernatural forces. While allopathic treatment was a remedy for some patients when traditional medicine failed, some parents explained that they did not receive proper treatment at modern health facilities for the conditions of their children, and they thought they got better care with the traditional healers. The use of Chinese medicine seemed to be increasing in the treatment seeking habits of the populations, according to our respondents, due to the lower costs compared to allopathic treatment and the effectiveness. Many of our respondents mentioned combining treatments as a reaction to a therapeutic failure or from a desire to quickly recover health.

The factors that legitimized this social practice mentioned were lack of financial means, geographical location of the health centers, and mismatch between supply and demand for treatment.

‘*It's a budget*, *others say that if you do not have a ½ million*, *you cannot heal*, *when you go there eh*, *so people prefer to go to the traditional healers*, *those*, *those*, *hey we are in a place even poverty has gained ground*, *this is what poses a problem*, *so people prefer to find their way among traditional healers*.*’ (Nurse*, *Ahouabo)*

‘*If we cannot afford to go to the hospital*, *we have to look for the healers next door*. *Down there when we go at least*, *it's a little less*, *with the leaves*, *well*, *if it works*, *that's it; if it does not work*, *we keep turning*.*’ (Parent*, *Bouapé****)***

With regard to leprosy and BU, people were not always well informed about the possibilities for care and cure of these diseases. Some healthcare providers noted that the modern treatment of these diseases is limited in that it deals only with curing of the infection while leaving the aesthetic aspects behind. Further, there were the limits to providing treatment for the disabilities caused.

‘*In principle*, *that's it*. *Leprosy with no disability*, *do not come here*, *they are taken care at other places*. *They receive chemotherapy and they leave*. *There if the persons present disability*, *they come here*. *When they come*, *I'm here*, *I do not see what we can do for its complications*. *That’s it*. *I say that the persons who come here with leprosy and disability*, *how we give them a goal*? *Treatment of disability is not at a good technical level*, *it poses a problem*.*’ [Doctor]*

### PART II: Results from the CDLQI questionnaire

A total of 184 schoolchildren (110 males, 74 females) aged between 5 and 15 completed the questionnaire (Table 1). The median age was 10 years (IQR 8–12). A total of 247 skin diseases were diagnosed and 48 children (26%) were diagnosed with more than two diseases (Table 1). In this case, the predominant diagnosis determined by severity was noted and used in the analysis. It took approximately 15 to 20 minutes for each child to complete the questionnaire with assistance by members of the survey team.

**Table 1:** Spectrum of skin diseases in schoolchildren in the CDLQI study.

	N	%
Total number of children in the CDLQI study	184	100.0%
Gender		
Boys	110	59.8%
Girls	74	40.2%
Age (years)		
5–8 years	50	27.2%
9–12 years	110	59.8%
13–15 years	24	13.0%
Total number of skin diseases diagnosed	241	100.0%
*Mycotic infection*		
Total	127	52.7%
*Pityriasis versicolor*	49	20.3%
*Tinea capitis*	56	23.2%
*Tinea pedis*	9	3.7%
*Tinea corporis*	11	4.6%
*Onychomycose*	2	0.8%
*Bacterial infection*		
Total	9	3.7%
*Folliculitis*	4	1.7%
*Abscess*	1	0.4%
*Impetigo contagiosum*	4	1.6%
*Buruli ulcer*	5*	2.0%
*Leprosy*	1^†^	0.4%
*Viral infection*		
Total	7	2.8%
*Molluscum contagiosum*	1	0.4%
*Warts*	3	1.2%
*Measles*	2	0.8%
*Varicella*	1	0.4%
*Parasitic infection*		
Total	15	6.1%
*Scabies*	15	6.1%
*Inflammatory skin disease*		
Total	52	21.1%
*Seborrheic dermatitis*	1	0.4%
*Acne vulgaris*	2	0.8%
*Eczema*	24	9.7%
*Acute prurigo*	12	4.9%
*Atopic dermatitis*	8	3.2%
*Pruritus*	5	2.0%
*Miscellaneous skin disease*		
Total	31	12.6%
*Wounds*	17	6.9%
*Miliaria / heat rash*	6	2.4%
*Vitiligo*	1	0.4%
*Angular cheilitis*	1	0.4%
*Localized hyperhidrosis*	3	1.2%
*Varicella scar*	3	1.2%
Number of skin diseases diagnosed per child		
1	136	73.9%
2	36	19.6%
3	9	4.9%
4	3	1.6%

* 4 new cases confirmed by PCR; 1 treated case with characteristic deformities

^†^ Multi-bacilli case confirmed by clinical signs and slit-skin smear

The CDLQI scores ranged from 0 to 29 with a median score of 5 (IQR 2–9). There was a trend toward higher median CDLQI scores in girls (5.5, IQR 3–9.25) than in boys (5, IQR 2–8), although the differences were not statistically significant (p = 0.25). There was also no significant difference in CDLQI scores across age groups (p = 0.29). Table 2 presents the CDLQI scores by stratified for severity. Forty-one participants (22%) expressed ‘no effect’, while 15 participants (8%) and 9 participants (5%) felt ‘very large effect’ and ‘extremely large effect’ due to their skin conditions, respectively. The latter included patients with BU (n = 4), leprosy (n = 1), scabies (n = 1), tinea capitis (n = 2), and generalized eczema (n = 1).

**Table 2:** CDLQI scores by stratified for severity.

CDLQI score	Severity group	No. of children	% of children
0–1	No effect	41	22%
2–6	Small effect	78	43%
7–12	Moderate effect	41	22%
13–18	Very large effect	15	8%
19–30	Extremely large effect	9	5%

**Fig 3:**
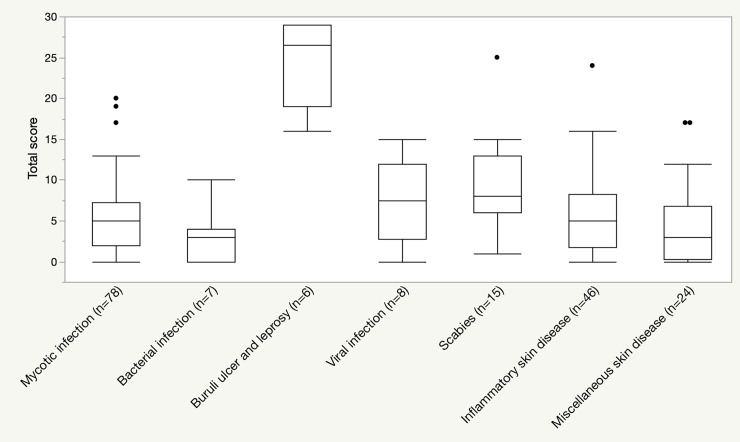
Distribution of CDLQI score by disease groups.

Fig 3 and Table 3 presents the distribution of CDLQI scores by disease groups and also by the six CDLQI dimensions. The scores were predominantly the highest for the BU and leprosy group for every dimension, followed by scabies. For scabies, the ‘symptom and feelings’ and ‘sleep’ dimensions were the most affected. For the other groups, aggregated data did not show a significant impact on the quality of life from the skin conditions. However, a wide range of scores was observed within the same disease group.

**Table 3:** Median and interquartile range for children’s dermatology life quality index (CDLQI) by the six dimensions.

	Symptom and feelings (0–6)	Leisure time activities (0–9)	School (0–3)	Personal relationships (0–6)	Sleep (0–3)	Treatment (0–3)	TOTAL (0–30)
Mycotic infection (n = 78)	2 (0–3)	1 (0–1.25)	0 (0–1)	0 (0–2)	0 (0–1)	0	5 (2–7.25)
Bacterial infection (n = 7)	2 (0–3)	0	0 (0–1)	0 (0)	0 (0–1)	0	3 (0–4)
Buruli ulcer and leprosy (n = 6)	5.5 (5–6)	9 (6–9)	1.5 (0–3)	5.5 (4.75–6)	3 (0–3)	3 (1.75–3)	26.5 (19–29)
Viral infection (n = 8)	3 (0.75–5)	1 (0.25–2.5)	0 (0–0.75)	1 (0.25–2.5)	1 (0–1.75)	0 (0–0.75)	7.5 (2.75–12)
Scabies (n = 15)	3 (2–4)	1 (1–3)	1 (0–2)	0 (0–3)	2 (1–3)	0	8 (6–13)
Inflammatory skin disease (n = 46)	2 (1–3)	1 (0–3)	0 (0–1)	0 (0–1)	0 (0–2)	0	5 (1.75–8.25)
Miscellaneous skin disease (n = 24)	1 (0–3)	1 (0–2)	0 (0–1)	0 (0–1)	0 (0–1)	0	3 (0.25–6.75)
ALL SKIN DISEASES (N = 184)	2 (1–3)	1 (0–2)	0 (0–1)	0 (0–1)	0 (0–1)	0	5 (2–9)

## Discussion

Skin diseases were ranked as one of the five leading causes of years lived with disability (YLDs) among the total 354 diseases and injuries from 1990 to 2017 [18]. This indicates that they can have a significant impact on the lives of those affected–limiting, for instance, their social participation. However, many studies investigating the impact of skin diseases have been performed in high-income countries, and the situation in the low- and middle-income countries where their prevalence is higher has seldom received attention. In the skin prevalence study that we performed at the same study site, one out of four children was found to be affected with some kind of skin disease, of which the distribution of skin diseases were similar to that reported here [13].

To shed more light on this neglected area, we have examined in this study the knowledge, attitudes, and practices towards skin diseases amongst children in rural Côte d’Ivoire and assessed the impact of skin diseases on their lives using a mixed-methods study design. We found that there was general ignorance or neglect of skin diseases–which had a large impact on the parents’ health seeking behavior. However, a range was observed in the extent to which skin diseases had an impact on children’s quality of life; sometimes there was a considerable impact. Nonetheless, the largest impact on the quality of life from the skin conditions was observed in children with BU and leprosy. This was a clear finding, but the CDLQI tool was useful in assessing the extent of how these children were affected by the disease.

We found that there was relatively a good recognition of skin diseases in our study population, both amongst children and adults, as they were often seen in the course of their everyday life. However, while children are often significantly impacted by the skin diseases (such as those manifesting with pruritus [scabies] and skin patches [tinea capitis] leading to bullying), they are often regarded by parents and guardians as insignificant since they don’t seem to have any mortality impact, leading to neglect and ignorance as well as low likelihood to seek medical assistance. Heukelbach *et al*. also highlighted this indifference towards common skin diseases in Brazil and in Nigeria, and attributed this feeling to intricate linkage to people’s daily life, *i*.*e*., as they are so common, they have become a part of their normal life [19, 20]. For those children who received treatment in our study, most did not go to hospitals or even to the traditional healers, but rather obtained remedies that were readily available at their homes or at the nearby pharmacies; this may be why the CDLQI score for treatment may have been very small for most skin diseases. On the other hand, we also need to acknowledge the challenges faced by these poor and remote communities with regards to limitations in access-to-care and availability of resources (finance, medicines, etc.), which further diminishes the motivation to seek care. This was found in our study population but it is the reality that prevails in most rural villages in sub-Saharan Africa [21].

Despite the general neglect and ignorance by the adults, variations were observed as to how the skin diseases had affected children in our findings from both the interviews and the CDLQI questionnaire applied to them. Similar findings were made in a meta-analysis on quality of life in children with skin diseases by Olsen *et al*. that most skin conditions in children have a ‘small effect’; however, the range is large and that actually a significant proportion of children with many common skin diseases experience a very large effect on quality of life [22]. Some children in our study confessed that bullying or rejection by their peers led to interference with their social relationships which lowered their self-esteem. This appears to have been the experience of a minority of participants in our survey, however, we need to acknowledge that there are children who can be affected badly by even the commonest skin diseases seen in these communities, such as tinea capitis, a finding which, to our knowledge, has not been documented before.

It is not difficult to imagine how pruritus can affect children’s quality of life, depriving them of good sleep or hindering their academic performance as well as the status of well-being–a situation described by some of the children with scabies and inflammatory skin diseases in this study. The CDLQI score for scabies was the second highest after BU and leprosy in our study, and showed particular impacts on children’s sleep and school performance. Our score for scabies was very similar to that reported from a study carried out in Ethiopia which reported a median score of 7 (IQR 6–9) [23]. Atopic dermatitis is another pruritic condition previously studied and known to be associated with high CDLQI score. In a meta-analysis by Olsen *et al*., a combined point estimate for CDLQI of patients with atopic dermatitis from 38 studies was 8.5 (95% confidence interval: 7.1–9.8, range: 0–29) [22]. We observed only a ‘small effect’ in children with atopic dermatitis in our study, but this may have been due to the limited severity of the disease that we encountered. Interestingly, Worth *et al*. reported that the feeling of shame was also associated largely in a decrement in the quality of life in patients with scabies, and therefore there is a need to look for consequences of these pruritic diseases beyond loss of sleep or reduced academic performances [24].

Detailed investigations of leprosy and BU was beyond the scope of this study as these are two big themes in their own right. However, we observed that preconceived concepts about leprosy and BU existed in the communities, which often led to feelings of stigma and discrimination which have been identified in many other past studies [25–32]. The local names given to the two diseases, “kokobé” (pity) for leprosy and “wépion” (amputated wound) for BU in our study were interesting as it reflected how the community people perceived these diseases. However, stigma is a complex phenomenon and can be only understood from evaluation of both sides, public stigma and self-stigma, which are interrelated and closely linked [27]. Self-stigma may foster isolation and could be the origin of a negatively self-reinforcing cycle, a tendency which was also observed by our study participants, and which needs further investigation [33, 34].

The myths around causes of leprosy, such as “blood”, “curse”, and “bad deeds”, are interestingly very similar among many endemic communities worldwide and were also observed in our study [25, 32, 29]. A study by Singh *et al*. found that good knowledge on leprosy transmission was positively associated with attaining favorable attitudes towards the disease in Nepal [25]. Indeed, in our study, perceived contagiousness rather than the physical appearance of their conditions seemed to have been more closely associated with the feeling of rejection. Providing them with a proper knowledge base, especially on the mode of transmission may play an essential role in reducing this feeling of rejection–and ultimately stigma and discrimination—towards leprosy. On the contrary, much of the rejection against patients with BU in our study was derived from its unique smell, or “stench”, a common observation also made in other studies of BU [35].

Our findings draw attention to the fact that there is need for good messaging strategies to be put in place in order to reach out to people in the community by providing them with knowledge on skin NTDs, including leprosy and BU, as these diseases are most often associated with stigma and discrimination [28]. While knowledge about common skin diseases among schoolchildren was derived from personal experience, knowledge about leprosy and BU often came from second-hand information from parents or teachers, and sometimes from the media. A study from Western Nigeria found that the health promotion messages about leprosy in primary school books continued to influence discriminatory ideas and practices towards leprosy, for a long time as well as those derived from the media [29]. It is crucial that children obtain the right information before these misperceptions become ingrained, in order to reduce stigma and discrimination towards those with leprosy and BU. Some teachers in our study associated skin diseases, especially leprosy and BU, with poor hygiene and also used these two diseases as examples in education on personal hygiene. This is likely to have had an effect on reinforcing stigma and discrimination against leprosy and BU in our study population.

There is a move toward integration of skin NTDs with some of the water, sanitation, and hygiene (WASH) activities–including education as outlined in the current WHO WASH guidelines [36]. As there was already recognition in the community that infectious skin diseases are associated with poor hygiene by and large, there is potential synergy. However, this needs to be carefully implemented as there may also be a risk of increasing stigma and discrimination or rejection of those with other skin diseases as well as leprosy and BU. Examples of good practice which still are scarce need to be gathered in order to develop an effective but also caring approach and to avoid such undesirable outcomes.

The high prevalence of skin disease in the community may make people indifferent to them and this affects their health seeking behavior, a finding also observed in our study [19, 20]. Often, delay in accessing healthcare facilities by patients with leprosy and BU are reported to occur due to a belief that these diseases are a result of witchcraft, fear of incurring cost for treatment (direct and indirect), or stigma and discrimination [37–39]. In addition, the asymptomatic nature of the initial stages of these diseases, and also a fact that there are so many other skin diseases in children to differentiate from, contributes to this failure to access healthcare facilities at an early stage. This is also described by Mulder *et al*. for BU in that the disease at an early stage is not considered serious enough thus leading to delay in detection [37]. Higher awareness towards skin diseases as a whole -not just leprosy or BU- is needed in order to change this health seeking behavior in the community, and to increase passive case finding for the more disabling diseases. As children with various skin diseases are affected by their diseases, as our findings suggest, such a holistic approach will also be beneficial to them and potentially increase the community acceptance of interventions.

The CDLQI questionnaire has been previously performed in a few African countries, including in patients with BU [23, 40]. However, this was the first study to adapt the cartoon version for use in African children with locally and culturally appropriate wording and images for this target population. The tool was found to be very useful in assessing the quality of life of children affected with skin diseases, enabling engagement of children. However, the time taken to complete the questionnaire was long, approximately 15 to 20 minutes for each child, and this limited the population sample size. This was a lot longer compared with the time given in the original study which was 90 seconds [15]. We believe that it would be necessary in any future surveys of this kind to devote a significant amount of time in order to elicit detailed and accurate data on the impact of skin diseases in this population, given barriers pertaining to level of education, literacy and cultural and language differences.

There are limitations to our study. Firstly, interviews were performed in French, by the trained sociologists, and not in the local dialect. Although French was the official language most people spoke in our study site, we may have missed some important information and may have created some bias. Secondly, we did not collect information on the severity of skin diseases for the CDLQI scores to ensure better interpretation of some results as the work was carried out as a pilot study, as mentioned earlier. However, the information may have enriched our study findings more by enabling an understanding of the relationships between the symptoms and the scores. Furthermore, many children presented with two or more skin diseases. This may have influenced the CDLQI scores although we attempted to identify the most predominant one. Thirdly, this study was conducted in one district in Côte d’Ivoire and therefore care needs to be taken in generalizing the results to a wider region. Nonetheless, this study stands significant in being one of the first to have attempted to determine the knowledge, attitudes, and practices as well as the impact of common skin diseases in schoolchildren, and further, to compare against skin NTDs (leprosy and BU) using a mixed methods study design. Use of interviews and CDLQI questionnaire was synergic and enabled us to achieve a better understanding of this important topic. Many of these findings have not been well documented in the past and we therefore believe they are of value.

In conclusion, the impact of common skin diseases in children was underestimated by both patients and those close to them, including parents, in a setting where leprosy and BU were endemic and where further disease control interventions for skin NTDs are planned. Awareness raising, or skin health promotion, which supports changing the health seeking behavior for skin conditions in general will aid in early detection and treatment of skin NTDs, and may also prove beneficial for those affected by other skin diseases. Although many adults seem to have been ignorant about the skin conditions in their children, experience together with good outcomes given may change their attitude, as with their children. Paying attention to the health problems faced by the community is likely to ensure community acceptance of skin NTD interventions and enhance community engagement. Educational opportunities, such as those associated with WASH initiatives, should be utilized to their utmost, but careful messages need to be developed and delivered, in order to avoid creating or increasing stigma and discrimination. Skin NTDs occur against a background of a high prevalence of common skin diseases, and therefore addressing these may become the key to success in the fight against skin NTDs.

## Supporting information

S1 Appendix Topic guidelines used in the study: ‘Impact of common skin diseases on children in rural Côte d’Ivoire with leprosy and Buruli ulcer co-endemicity: a mixed methods study’. (DOC)

S1 Fig Modified Children’s Dermatology Life Quality Index (CDLQI) questionnaire used in the study [French version]. (PNG)

S1 Table Process of developing the modified Children’s Dermatology Life Quality Index (CLDQI) questionnaire from the original English version into French. (DOCX)

S2 Table Maximum sampling quotas and percentage of participants who were recruited for the top five common skin diseases. (DOCX)
