# Supplementary material for: Impact of common skin diseases on children in rural Côte d’Ivoire with leprosy and Buruli ulcer co-endemicity: A mixed methods study
Source: PLoS Negl Trop Dis. 2020 May 18;14(5):e0008291. doi: 10.1371/journal.pntd.0008291 (PMC7274456; doi:10.1371/journal.pntd.0008291)
Supplement: S1 Appendix — (DOC) [file pntd.0008291.s001.doc]

**TOPIC GUIDELINES FOR FOCUS GROUP DISCUSSIONS**

**WITH STUDENTS IN PRIMARY SCHOOLS**

- Welcome
- Introduction of the interviewer(s)
- Informed consent informed assent

LIFE QUALITY OF CHILDREN WITH SKIN PROBLEMS IN PRIMARY SCHOOLS

- How do you feel about your skin problems?
- How were you treated because of your skin problems?
- For those of you who got treatment, which treatment did you receive? How did you feel about receiving the treatment?
- To which extent, your skin problem affected your outings, your games, your sport activities with friends?
- To which extent, your skin problem affected your school work?
- To which extent, your skin problem affected your relationship with your family, friends and community?
- What are the diseases that you know that are in your community?

LEPROSY (USE LOCAL NAME IF APPROPRIATE)

- What do you know of leprosy?
- Where did you hear about it? Who talked about it?
- How can we catch it?
- Who can get leprosy? Why?
- How do you recognize a leprosy patient?
- What do you think about leprosy?
- How does your family see leprosy? What do people say about leprosy in your community?
- How do you call leprosy in your mother tongue? What is the meaning?
- What are the consequences of leprosy?
- What will be your reaction if one of your friends is a leprosy patient?

BURULI ULCER (USE LOCAL NAME IF APPROPRIATE)

- What do you know about Buruli ulcer?
- Where did you hear about it? Who talked about it?
- How can we catch it?
- Who can get Buruli ulcer? Why?
- How do you recognize a Buruli ulcer patient?
- What do you think about Buruli ulcer?
- How does your family see Buruli ulcer? What do people say about Buruli ulcer in your community?
- How do you call Buruli ulcer in your mother tongue? What is the meaning?
- What are the consequences of Buruli ulcer?
- What will be your reaction if one of your friends is a Buruli ulcer patient?

HEALTH SEEKING BEHAVIOUR

- Where do people of your community go to get cure when they get these diseases?
- According to you, where should we go for treatment when we get these diseases? Why?

**TOPIC GUIDELINES FOR FOCUS GROUP DISCUSSIONS WITH PARENTS**

**TOPIC GUIDELINES FOR INTERVIEWS WITH LEADERS**

- Welcome
- Introduction of the research study and the interviewer(s)
- Informed consent

*Questions:*

1. What are the common skin problems you see in children in your village? Please provide local terms for the conditions.
2. Where do you/families take your/their children to seek for care/treatment when they have skin problems?
3. What do you think are the barriers for children visiting healthcare facilities for their skin problems?
4. What are the barriers for children to continuing treatment for their skin problems?
5. What do you think are the demands & needs for improving life of children with skin problems?
6. In what way do you see stigma & discrimination among children towards skin problems? Please provide examples.
7. What do you know about leprosy?
8. What do you know about Buruli ulcer?
9. How are these two diseases regarded among the community members? [*Check if regarded as common diseases or not.*]
10. What do you think are the demands & needs for these two diseases in your community?
11. In what way do you see stigma & discrimination among children towards leprosy / Buruli ulcer?

**TOPIC GUIDELINES FOR SCHOOLTEACHERS**

- Welcome
- Introduction of the research study and the interviewer(s)
- Informed consent

*Questions:*

1. What are the common skin problems you see in children in your class? Please provide local terms for the conditions.
2. Do you have in your programme classes related to skin problems? If yes, how do you teach children? What is the component?
3. Do you have any children in your class undergone/undergoing any treatment for skin problems? If yes, for what symptoms or diseases?
4. Where do parents take the children go seek for care/treatment when they have skin problems? Why?
5. For you, why children don’t go to health center when they have skin problems?
6. What do you think are the demands & needs for improving life of children with skin problems?
7. Do you see stigma & discrimination among children in your class with skin problems? If yes, in what way.
8. What do you know about leprosy? Are there any occasions you talk to your class about leprosy? If yes, what do you teach children about leprosy? Have you ever been asked about leprosy by a child? If yes, what do they want to know?
9. What do you know about Buruli ulcer? Are there any occasions you talk to your class about Buruli ulcer? If yes, what do you teach children about Buruli ulcer? Have you ever been asked about Buruli ulcer by a child? If yes, what do they want to know?
10. What do you think are the demands & needs for these two diseases in your community?
11. In what way do you see stigma & discrimination among children towards leprosy / Buruli ulcer?

**TOPIC GUIDELINES FOR HEALTHCARE WORKERS**

**(COMMUNITY HEALTH WORKERS / NURSES / DOCTORS)**

- Welcome
- Introduction of the research study and the interviewer(s)
- Informed consent

*Questions:*

1. What are the common skin problems you see in children in your community? Please provide local terms for the conditions.
2. What kind of advice do you provide to children and to parents of such children?
3. What are the reasons why children not go when they have skin problems?
4. What is the explanations for stopping of recommended treatment given by nurses?
5. What do you think are the demands & needs for improving quality of life of children with skin problems?
6. Do you see stigma & discrimination among children in your class with skin problems? If yes, in what way. Why?
7. What do you know about leprosy?
8. What is the cause of leprosy?
9. What is your role in leprosy elimination programme?
10. What do you know about Buruli ulcers?
11. What is the cause for this disease?
12. What is your role in Buruli ulcer control programme?
13. How are these two diseases regarded among the community members?
14. What do you think are the demands & needs for these two diseases in your community?
15. In what way do you see stigma & discrimination among children towards leprosy / Buruli ulcer?

**TOPIC GUIDELINES FOR TRADITIONAL HEALERS**

- Welcome
- Introduction of the research study and the interviewer(s)
- Informed consent

*Questions:*

1. What are the common skin problems you see in children visiting you? Please provide local terms for the conditions. Estimate of number of patients per day/ per week/ per month.
2. What do you think are the causes of these diseases?
3. What kind of treatments do you provide to such children? Please give details of your treatments.
4. What do you think are the reasons for children not visiting public healthcare facilities for their skin problems?
5. What is the clinical course of these children?
6. What are the barriers for children to continuing treatment for skin problems?
7. What do you think are the demands & needs for improving quality of life of children with skin problems?
8. In what way do you see stigma & discrimination among children with skin problems? Please provide examples.
9. What do you know about leprosy? What kind of treatments do you provide to patients with leprosy? Have you ever advised the patient to visit health facilities / clinics for their conditions? Why or why not?
10. What do you know about Buruli ulcer? What kind of treatments do you provide to patients with Buruli ulcer? Have you ever advised the patient to visit health facilities / clinics for their conditions? Why or why not?
11. How are these two diseases regarded among the community members? [*Check if regarded as common diseases or not.*]
12. What do you think are the demands & needs for these two diseases in your community?
13. In what way do you see stigma & discrimination among children towards leprosy / Buruli ulcer?
