# Supplementary figures and images for: Impact of common skin diseases on children in rural Côte d’Ivoire with leprosy and Buruli ulcer co-endemicity: A mixed methods study
Source: PLoS Negl Trop Dis. 2020 May 18;14(5):e0008291. doi: 10.1371/journal.pntd.0008291 (PMC7274456; doi:10.1371/journal.pntd.0008291)

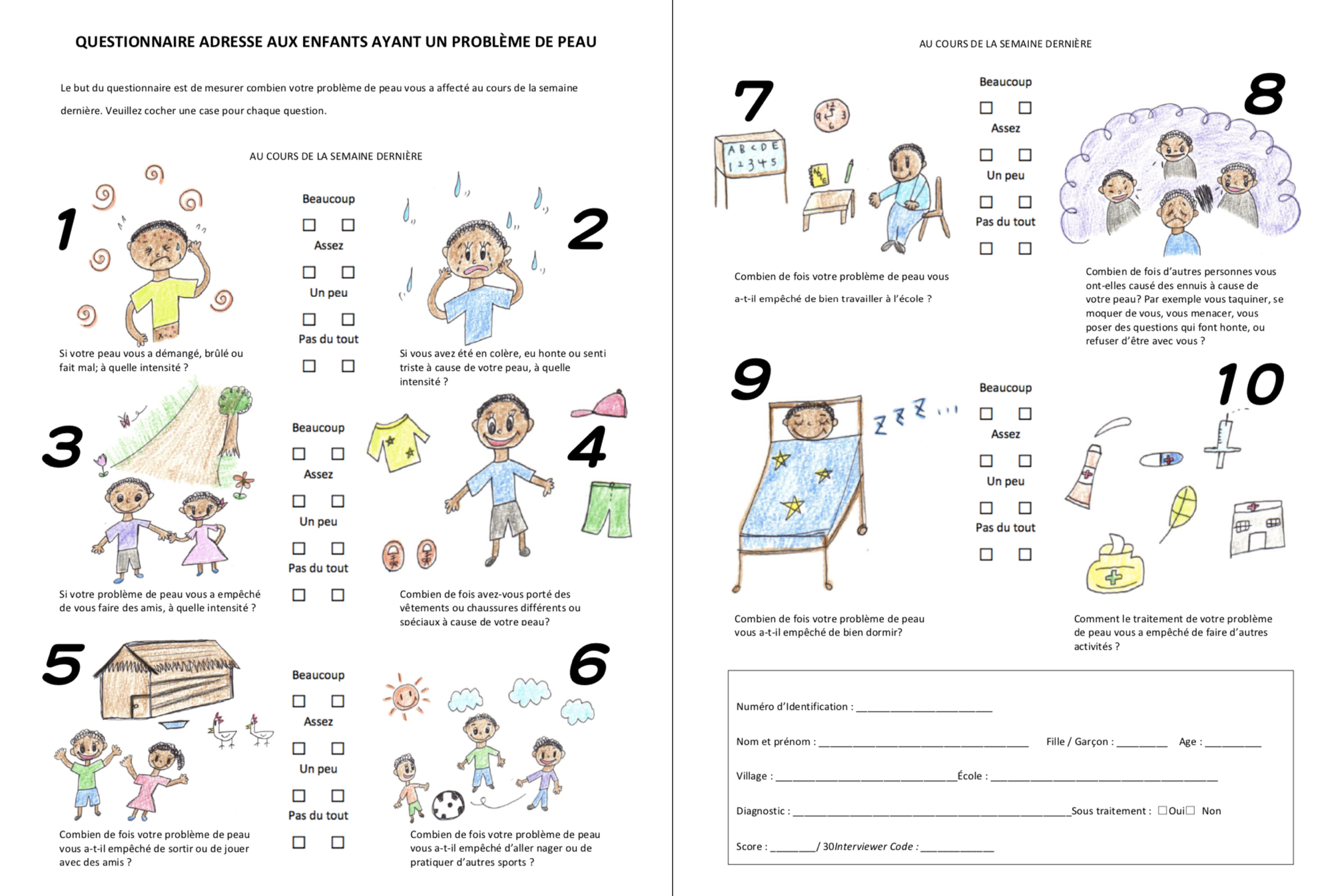

Supplement: S1 Fig — (PNG) [file pntd.0008291.s002.png]
