# Supplementary material for: Impact of common skin diseases on children in rural Côte d’Ivoire with leprosy and Buruli ulcer co-endemicity: A mixed methods study
Source: PLoS Negl Trop Dis. 2020 May 18;14(5):e0008291. doi: 10.1371/journal.pntd.0008291 (PMC7274456; doi:10.1371/journal.pntd.0008291)
Supplement: S1 Table — (DOCX) [file pntd.0008291.s003.docx]

**Table S1. Process of developing the modified Children’s Dermatology Life Quality Index (CDLQI) questionnaire in French from the original English version**

The table shows a comparison between the already-validated original questions in the English version [Lewis-Jones MS, Finlay AY. The Children’s Dermatology Life Quality Index (CDLQI): initial validation and practical use. BJD 1995; 132(6): 942-949] and the modified tool in French that we developed for use in our study. The first set of draft questions were prepared which were pilot-tested in CE4 level (Grade 4) schoolchildren of Abidjan, an economic capital city of Côte d’Ivoire, with skin diseases. The tool was tested repeatedly to different children by the two sociologists in our study (CCC and ATG) until it reached to a point where the children were understanding the questions with less confusion and took less time for them to complete the questionnaire, *i.e.*, the questions were translated in a linguistically and culturally accepted way. A total of 30 children were interviewed during this process. The final set of questions is provided in the right column.

|  | **Original questions [English]** | **Draft questions [French]** | **Final questions [French]** |
| --- | --- | --- | --- |
| Q1 | How itchy, ‘scratchy’, sore or painful has your skin been? | À quel point avez-vous eu des démangeaisons, “gratté”, lésions ou des douleurs? | Si votre peau vous a démangé, brûlé ou fait mal; à quelle intensité? |
| Q2 | How upset or embarrassed, self-conscious or sad have you been because of your skin? | À quel point avez-vous été bouleversé ou embarrassé, gêné ou triste à cause de votre peau? | Si vous avez été en colère, eu honte ou senti triste à cause de votre peau, à quelle intensité? |
| Q3 | How much has your skin affected your friendships? | Est-ce que vos amis ou camarades sont partis (ne jouaient plus ou ne causaient plus) à cause de votre problème de peau? | Si votre problème de peau vous a empêché de vous faire des amis, à quelle intensité? |
| Q4 | How much have you changed or worn different or special cloths / shoes because of your skin? | Combien de fois par jour vous deviez changer vos habits ou vos chaussures à cause de votre problème de peau ? | Combien de fois avez-vous porté des vêtements ou chaussures différents ou spéciaux à cause de votre peau? |
| Q5 | How much has your skin trouble affected going out, playing or doing hobbies? | Dans quelle mesure vos problèmes de peau ont-ils affecté les sorties, les loisirs ou les hobbies? | Combien de fois votre problème de peau vous a-t-il empêché de sortir ou de jouer avec des amis? |
| Q6 | How much have you avoided swimming or other sports because of your skin trouble? | Combien avez-vous évité la natation ou d'autres sports à cause de vos problèmes de peau? | Combien de fois votre problème de peau vous a-t-il empêché d’aller nager ou de pratiquer d’autres sports? |
| Q7^＊^ | How much did your skin affect your school work? | À quel point vos problèmes de peau ont-ils joué sur votre travail scolaire? | Combien de fois votre problème de peau vous a-t-il empêché de bien travailler à l’école? |
| Q8 | How much trouble have you had because of your skin with other people calling you names, teasing, bullying, asking questions or avoiding you? | À quel point avez vous eu honte des surnoms qu’on vous donnait à cause de votre problème de peau? Ou à quel point vos amis se moquaient de vous ou vous fuyaient à cause de votre problème de peau? | Combien de fois d’autres personnes vous ont-elles causé des ennuis à cause de votre peau? Par exemple vous taquiner, se moquer de vous, vous menacer, vous poser des questions qui font honte, ou refuser d’être avec vous? |
| Q9 | How much has your sleep been affected by your skin problem? | À quel point votre problème de peau vous a-t-il empêché de dormir? | Combien de fois votre problème de peau vous a-t-il empêché de bien dormir? |
| Q10 | How much of a problem has the treatment for your skin been? | Est-ce que vous ou vos parents aviez cherchés une aide extérieure pour soigner votre problème de peau? | Comment le traitement de votre problème de peau vous a empêché de faire d’autres activités? |

* The original version by Lewis-Jones *et al.* had two questions for Question 7. Listed here is Question 7a, and we decided to delete Question 7b: ‘If holiday time, how has your skin problem interfered with your holiday plans?’ based on the uncommon practice of people of Côte d’Ivoire going for vacation for their holiday time.
