# Supplementary material for: Impact of common skin diseases on children in rural Côte d’Ivoire with leprosy and Buruli ulcer co-endemicity: A mixed methods study
Source: PLoS Negl Trop Dis. 2020 May 18;14(5):e0008291. doi: 10.1371/journal.pntd.0008291 (PMC7274456; doi:10.1371/journal.pntd.0008291)
Supplement: S2 Table — (DOCX) [file pntd.0008291.s004.docx]

**Table S2. Maximum sampling quotas and percentage of participants who were recruited for the top five common skin diseases.**
